# Surface Modification and Stabilization of Eutectic Gallium Indium Nanoparticles with an Electrochemically Active Ligand Using Low Molecular Weight Phosphorothioates in Water

**DOI:** 10.1021/acsomega.5c02237

**Published:** 2025-06-11

**Authors:** José Catalán-Toledo, Jordi Romero-Pallejà, Núria Crivillers

**Affiliations:** 54449 Institut de Ciència de Materials de Barcelona (ICMAB-CSIC), Campus de la UAB s/n, Bellaterra 081093, Spain

## Abstract

Liquid metal nanoparticles (LM NPs) have attracted significant attention owing to their unique properties and wide range of potential applications from electronics to biomedicine. In this study, we investigate the surface chemistry of eutectic gallium indium (EGaIn) NPs using different phosphonic acid derivatives in aqueous media. Our findings show that EGaIn NPs exhibit significant surface chemical composition changes when exposed to these ligands in solution during the ultrasonication-assisted synthesis in water, displaying optimal long-term colloidal stability in alkaline media. Interestingly, it is demonstrated for the first time that the use of sulfur-containing phosphonic acids, particularly phosphorothioates, leads to improved colloidal suspension stability and higher control over the NP size distribution. In addition, using a phosphorothioate derivative with a terminal *N*-hydroxysuccinimide group, a novel synthetic conjugation strategy to incorporate redox-active molecules, in particular ferrocene, onto the NP surface has been developed, permitting broadening of their potential applications in (electro)­catalysis and sensing.

## Graphical abstract

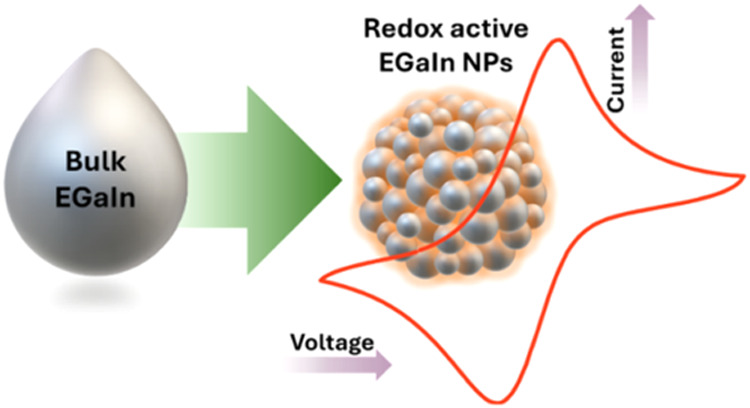

## 1 Introduction

Liquid metals (LMs) can be considered key players in the future of functional applied materials.^1,2^ They have attracted much interest because they combine metallic and fluidic properties making them suitable as electrodes in advanced printed flexible electronic devices, with extraordinary market prospects.^3^ Currently, one of the most employed LMs is the eutectic gallium indium (EGaIn) alloy, which is composed of 75.5% gallium and 24.5% indium, displaying a high electrical conductivity and attractive rheological properties. Some of its main characteristics are low melting point, relatively high density, and low toxicity, due to its negligible vapor pressure, making it safer compared with other LMs such as mercury.^4^ The surfaces of pure Ga or EGaIn alloys are easily oxidized, leading to the spontaneous formation of an amorphous gallium oxide layer (0.7–3 nm, mainly Ga_2_O_3_) even in low oxygen concentrations.^5,6^ Along with the research conducted with LMs in bulk, the preparation of micro/nanosized liquid metal particles (LMP) has awakened much interest. The decrease in size and the atomic rearrangement due to the increased surface curvature lead to different electrical/thermal conductivities, reactivity, optical, and melting points compared with bulk properties. They have already been explored as appealing materials for catalysis, energy harvesting, and biomedicine purposes.^7^ In electronics, the native oxide layer mentioned above can limit their application; however, the sintering of these NPs has proven to be an effective strategy for forming conductive electrical pathways.^8^ Furthermore, EGaIn NPs exhibit unique optical properties that differ from those of their bulk LM form. Unlike gold (Au) and silver (Ag) NPs, which have plasmonic responses in the visible-IR range, EGaIn NPs dispersed in ethanol show a broad plasmonic response from UV–visible, due to Ga large band gap (∼4.8 eV).^7,9,10^ Catalan-Gomez et al.^11^ theoretically showed that the width of the oxide shell encapsulating the metallic core of NPs is a crucial factor in controlling the plasmonic response. The oxide skin encapsulates the liquid core and decreases the surface tension of the liquid metal and helps to retain the shape. The encapsulation depends largely on several factors such as particle size, oxygen saturation in the solvent, and temperature during the synthesis. The uncontrolled growth of the oxide layer might limit the use of these particles in certain applications.^12–15^ This native oxide has been described as amorphous or poorly crystalline,^16^ highly smooth,^17^ and it is highly dependent on the surrounding environment, especially in an aqueous medium.^12,18,19^ The improvement of colloidal stabilization has been achieved using carbonated ions as **·**OH radical scavengers,^20^ but often relies on surface functionalization, which has been achieved employing small molecules, polymers, and biomolecules. Inspired by the well-known functionalization chemistry of other oxide NPs, similar anchoring groups such as amines,^12,21–23^ carboxylic acids,^24,25^ cathecols,^26–28^ thiols,^5,29^ trithiocarbonates,^30^ silanes,^31^ and phosphonic acids have been used for EGaIn particles.^32,33^ The surface modification allows to tune different electrical/thermal conductivities, reactivity, and optoelectronic properties compared with the bulk material, opening up to applications such as solution-processed composites, dynamic nanostructures, conductive inks, and plasmonic systems.^7,34,35^ Besides the tremendous progress and interest in LM-based materials for technological applications, they still face some stability limitations due to incomplete surface functionalization or the continuous formation, deformation, and cracking of the oxide skin. Thus, achieving precise control over the particle properties synthesized through a top-down approach remains a challenge in the field.

In this work, the ultrasonication of EGaIn bulk droplets^36^ was carried out to turn them into EGaIn nanosized particles. The oxide surface modification was achieved by the addition of the ligands during the sonochemical synthesis^27,37–41^ or by subsequent post-functionalization steps. Furthermore, effort was also put into developing a green synthetic protocol using water as a solvent avoiding organic solvents, such as dichlorobenzene^5,42^ and toluene.^43,44^ This study demonstrates that by strategically employing various phosphonic acid derivatives and precisely controlling the solution pH, we effectively mitigated the high reactivity of Ga and O_2_ in water, preventing the formation of undesired species during ultrasonic reactions.^35^ For that, we employed the octadecylphosphonic acid (ODPA), which is known to form self-assembled monolayers (SAMs) on metal-oxide surfaces,^45^ and S-octadecyl-O,O-dihydrogen phosphorothioate (SODPT) that has a higher solubility in aqueous media (Figure 1a). To the best of our knowledge, phosphorothioates have not been used as ligands for LM NPs and have barely used for other nanomaterial functionalization. In addition, to confer additional functionalities to EGaIn NPs, 2,5-dioxopyrrolidin-1-yl-11-(phosphorothio) undecanoate (2.5DPPNT, Scheme S1) bearing an NHS ester (*N*-hydroxysuccinimide) was employed to react with primary amines, here an aminoferrocene, leading to redox-active EGaIn NPs.

**Figure 1:**
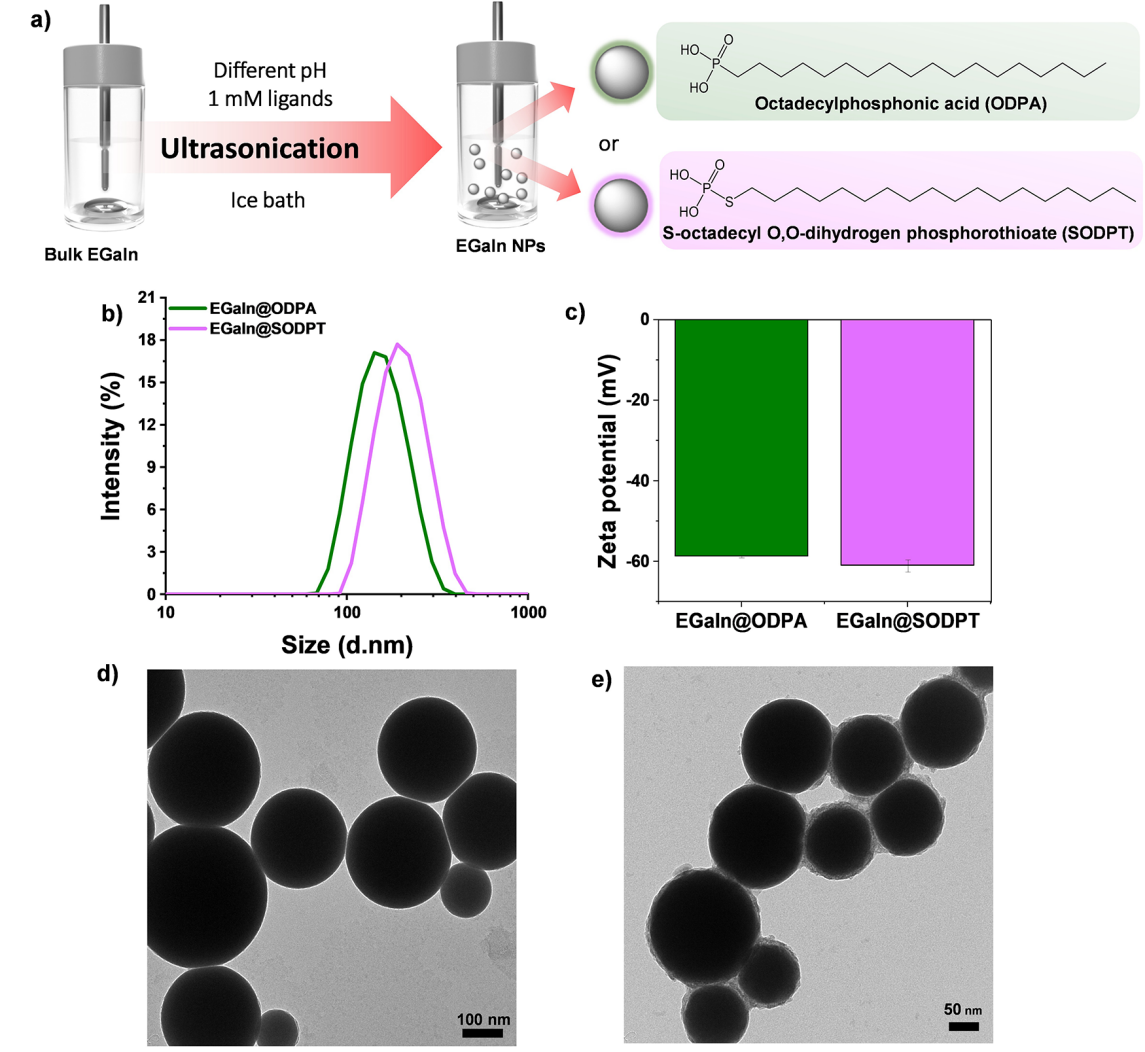
EGaIn NPs synthesized with phosphonic acid derivatives in an alkaline pH environment. (a) (right) Scheme of EGaIn NPs synthesis by ultrasonication method and (left) molecular structure of the ligands employed (ODPA and SODP) for the NP surface modification. (b) Hydrodynamic diameter distribution of EGaIn@ODPA and EGaIn@SODPT NPs. (c) Zeta-potential values for both samples. TEM images of (d) EGaIn@ODPA and (e) EGaIn@SODPT NPs.

## 2 Experimental Section

### 2.1 Materials

All chemicals and materials were used without further purification. EGaIn (75% Ga–25% In), aminoferrocene (Fc-NH_2_), acetonitrile, sodium hydroxide (NaOH), hydrochloric acid (HCl 37%), and tetrabutylammonium hexafluorophosphate (TBAFP_6_) were purchased from Sigma-Aldrich. ODPA from ABCr GmbH, SODPT, and 2.5DPPNT were obtained from Prochimia Surfaces.

### 2.2 EGaIn NPs Synthesis

Five mL of 1 mM solution of each ligand were prepared in ultrapure Milli-Q (MQ) water with the pH adjusted with minimal amounts of HCl (1 M) or NaOH (1 M) until pH 3 or pH 8, respectively. The solutions were vortexed and subjected to ultrasonication to facilitate dissolution. The solubility of SODPT was higher than that of ODPA. The solutions were then poured onto 100 mg of EGaIn placed in a 15 mL Falcon tube. Then, the tube was sealed, and a 3 mm tapered ultrasonication tip was placed 3–5 mm above the EGaIn drop. The mixture was then ultrasonicated using the Vibra Cell VC 505 probe sonicator (Sonics and Material Inc., Newtown, CT) at 22% amplitude for 40 min with 10 s on/off cycles in an ice bath. After the reaction, the solution was left undisturbed for 1 h, to let all of the bigger fragments sediment. After, to proceed with the purification process, the resulting suspension was first centrifuged at 1200 rpm (Frontier 5000 Multi Pro FC5718R, OHAUS) for 3 min to remove remaining big fragments and particles and then two further times at 5000 rpm for 5 min washing them with fresh Milli-Q water adjusted to the corresponding pH. To obtain smaller size NP fractions, the synthesized EGaIn NPs, after the first centrifugation at 5000 rpm, the supernatant was dialyzed using a cellulose membrane (D9652–100FT, Sigma-Aldrich) to remove traces of unreacted ligand for 72 h under constant stirring at room temperature, changing the adjusted alkaline water at least 3 times per day.

### 2.3 EGaIn NPs Post-functionalization Reactions

For the functionalization of EGaIn with 2.5DPPNT (Figure S14), 5 mL of 1 mM of 2.5DPPNT was ultrasonicated with the bulk EGaIn following the conditions described in the previous section. For the strategy shown in Scheme S2, 5 mL of a mixture of 2.5DPPNT and Fc-NH_2_ (1 mM/0.5 mM) in a 2:1 molar ratio was stirred for 30 min in an ice bath, and after this time freshly prepared EGaIn@SODPT NPs were added to the mixture, which was stirred for 1 h at room temperature. For the strategy shown in Scheme S3, 5 mL solution of 2.5DPPNT and Fc-NH_2_ (1 mM each) in a 1:1 molar ratio was mixed with freshly prepared EGaIn@SODPT NPs and stirred for 1 h in an ice bath. All of the obtained NPs were purified as previously described. All of the reactions were set maintaining the pH at a value around 8.

### 2.4 EGaIn NPs Characterization

Size distribution and stability over time were measured using dynamic light-scattering (DLS) and zeta-potential measurements were performed using a Zetasizer Nano ZS (Malvern) with backscatter detection (173°), controlled by Dispersion Technology Software (DTS 8.02, Malvern). The samples were also characterized by transmission electron microscopy (TEM), by drop casting 10 μL of concentrated NP suspensions onto a copper (Cu) or gold (Au) grid, using a JEM-1210 (JEOL) operating at 120 kV. High-resolution and line scan analysis were performed with high-angle annular dark-field scanning electron microscopy (HAADF-STEM) images acquired with an FEI Tecnai F20 (S)­TEM operated in both TEM and STEM modes at 200 kV, equipped with an EDAX super ultrathin window (SUTW) X-ray detector. Also, an FEI Magellan 400 L XHR was used for TEM characterization. Infrared measurements were performed by attenuated reflection Fourier transform infrared (ATR FT-IR) spectroscopy, using JASCO 4700LE. X-ray photoelectron spectroscopy (XPS) measurements were performed at room temperature with a SPECS PHOIBOS 150 hemispherical analyzer (SPECS GmbH) with a base pressure of 5 × 10^–10^ mbar using monochromatic Al Kα radiation (1486.74 eV) as an excitation source operated at 300 W. The energy resolution as measured by the full width at half-maximum (fwhm) of the Ag 3d_5/2_ peak for a sputtered silver foil was 0.62 eV. UV–vis measurements were performed in a JASCO V-780 spectrophotometer, using quartz cells with a 10 mm optical path for all measurements in solution. For electrochemical characterization, cyclic voltammetry (CV) analysis was performed in a three-electrode commercial glass electrochemical cell using an AUTOLAB 204 Potentiostat with NOVA 2.1.6 software. NPs were deposited onto a previously polished and electrochemically cleaned glassy carbon disc electrode (GCE) as a working electrode (0.5 cm diameter), with Ag/AgNO_3_ as a reference electrode and platinum wire as a counter electrode. A 0.1 M TBAPF_6_ (0.1 M) in CH_3_CN was used as the supporting electrolyte.

## 3 Results and Discussion

### 3.1 EGaIn NPs Synthesis and Characterization

The NP preparation was carried out by ultrasonication under different pHs, from acidic, near neutral, and alkaline.^44^ In strong acidic conditions (pH < 3), the oxide layer is partially dissolved, releasing Ga^3+^ ions and making surface modification challenging, though phosphonic acid groups can still bind to exposed Ga native oxide. In mild acidic conditions (pH 3–5), the oxide layer degrades slower, allowing weak interactions with phosphonic acids. In neutral to slightly alkaline conditions (pH ∼ 7–9), the oxide layer remains stable, promoting more uniform surface modification and better control over NP dispersion. However, in strong alkaline conditions (pH > 11), the Ga oxide layer reacts with hydroxide ions, forming Ga hydroxide species and compromising surface stability, also making it difficult for phosphonic ligands to bind effectively. In addition to pH, temperature is also a critical parameter. It has been shown that elevated temperatures lead to the formation of diverse Ga species such as GaOOH rods and different morphologies.^12^ For the experiments carried out in this study, the reaction vessel was always kept in an ice bath (Experimental Section).

First, to underscore the critical role of pH in nanoparticles formation, the synthesis of EGaIn NPs was conducted at varying pH values in the absence of any ligand. These experiments revealed an enhanced colloidal stability under alkaline conditions (Figure S1a). This conclusion is strongly supported by DLS measurements, zeta-potential (ζ) values, and TEM imaging. DLS measurements revealed that a better size control and lower polydispersity index (PDI) (149.6 ± 4.1 and 0.16 ± 0.01, respectively) were achieved in alkaline environments in comparison with acidic (356.6 ± 29.5 and 0.27 ± 0.01) or neutral conditions (1035 ± 117 and 0.42 ± 0.06) (Figure S1b). In addition, positive zeta-potential (ζ) values of +38.3 ± 5.2 mV and +25 ± 0.46 mV were measured for the latter two, precipitating a few hours after sample preparation. On the contrary, in alkaline conditions, the obtained ζ value was −54.4 ± 2.5 mV indicating higher colloidal stability (Figure S1c). This is in accordance with the fact that in alkaline media, the deprotonation of −OH groups at the oxide surface is favored, leading to a higher negative surface charge (i.e., more negative zeta potential) that leads to stronger electrostatic repulsion between particles reducing the tendency of aggregation. Morphological and structural analysis was performed by TEM, observing that both acidic and neutral conditions lead to the formation of non-spherical agglomerates. Meanwhile, alkaline environment led to more spherical-shaped structures (Figure S1d–f), although with a high sedimentation rate, in agreement with the DLS measurements. The observed poor stability may stem from the ultrasonic rupture of bulk EGaIn that promotes the dynamic formation of a fresh thin oxide layer as LM undergoes repeated breaking/reencapsulation under environmental O_2_ exposure. The absence of a ligand stabilizer contributes to particle aggregation resulting in limited long-term stability even in favorable pH conditions.

Following, ODPA and SODPT (1 mM in aqueous solution) were employed to prepare EGaIn@ODPA and EGaIn@SODPT NPs, respectively (Figure 1). The presence of R-PO­(OH)_2_ or R-S-PO­(OH)_2_, as anchoring groups, and the C_18_ alkyl chain give to these molecules a surfactant-like behavior, helping to stabilize NP dispersions (Figure 1a). In the case of acidic and neutral pH conditions, the formation of either aggregates or non-spherical-shaped particles (Figure S2) was observed for both ligands by DLS. On the contrary, narrower size distribution and lower PDI were obtained for synthesized NPs in alkaline conditions (pH = 8), with values of *d* = 127.9 ± 2.2 nm, PDI: 0.1 ± 0.02 for ODPA and *d* = 154.3 ± 4.1 nm, PDI: 0.036 ± 0.009 for SODPT (Figure 1a). Also, the zeta-potential values, which provide insight into surface charge and electrostatic repulsion, key factors in particle aggregation and stability, were measured to be −58.7 ± 0.3 mV and −61.0 ± 1.5 mV for ODPA and SODPT, respectively (Figure 1b), indicating the stability of both colloidal suspensions. In addition, the conductivity of the colloidal suspensions remained low and relatively stable over 54 days, with only slight decreases observed for both EGaIn@ODPA and EGaIn@SODPT systems, from 0.151 ± 0.005 mS/cm (day 1) to 0.130 ± 0.013 mS/cm (day 54), and from 0.152 ± 0.004 mS/cm (day 1) to 0.103 ± 0.002 mS/cm (day 54), respectively. This indicates minimal changes in electrolyte concentration, supporting that the measured ζ potential and DLS values reflect the intrinsic properties of the dispersed NPs.

In alkaline conditions, the phosphonic acid group is fully deprotonated (p*K*_a1_ ∼ 1.41–3.9 and p*K*_a2_ = 6.7–8.42 ^46^); however, in acidic and neutral conditions, this group is only partially deprotonated and the interactions with the oxide shell could be mainly through hydrogen bonds or electrostatic interactions, which do not provide a strong binding between the ligands and the surface during ultrasound-assisted synthesis. In addition, at pHs in the range of 6.5–9, Ga_2_O_3_ is relatively stable and in the presence of deprotonated phosphonic groups, the phosphoryl oxygen coordinates with the metal-oxide surface, followed by a heterocondensation reaction,^47^ promoting the formation of a ligand shell that stabilizes NPs.

For both samples, EGaIn@ODPA and EGaIn@SODPT NPs, STEM images revealed that NPs have predominantly spherical and well-defined shape, and the image analysis (*n* > 100 particles) yielded average particle diameters of 130.5 ± 32.2 nm for EGaIn@ODPA and 121.4 ± 34.1 nm for EGaIn@SODPT, as shown in the histograms in Figure S3. These values differ slightly from those obtained by DLS, as expected, considering that DLS measures the hydrodynamic diameter. Further, TEM images showed spherical NPs with a smooth surface and a ligand shell of around 3 nm for EGaIn@ODPA NPs (Figure 1d) and a rougher encapsulating shell of around 4–7 nm in the case of the EGaIn@SODPT NPs (Figure 1e). This was also observed by scanning electron microscopy (SEM) that showed a more wrinkle-type surface when using SODPT (Figure S4). Although the exact mechanism of the layer formation is not fully understood when employing SODPT, given that thiols (−SH) can react with Ga,^5^ it is likely that a dual coordination is taking place, both O and S bind to oxidized Ga^3+^ (via P–O–M bonds) and metallic Ga (via S–M bonds), respectively, during ultrasonication, increasing the ligand–surface interactions. Furthermore, sulfur may mediate the cross-linking with Ga^3+^ (released during ultrasonication) via S–Ga–S bridges, together with the hydrogen bond formation between −OH groups, creating a multilayered supramolecular structure. This leads to the formation of a shell composed of a Ga_2_O_3_ layer, with similar thickness to that of EGaIn@ODPA NPs, further encapsulated with a more complex hybrid shell, integrating both Ga_2_O_3_ and the chemically bonded ligand network.

High-resolution (HR) TEM, high-angle annular dark-field (HAADF), and energy-dispersive X-ray spectroscopy (EDX) were used to further characterize NPs. For EGaIn@ODPA NPs (Figure S5a), HRTEM images displayed in Figure S5b clearly show an organic layer with an average thickness of approximately 3 nm. Further, the HAADF/EDX scan line analysis (Figure S5c) confirmed the presence of Ga, In, O, and P over this region. As mentioned above, in the case of EGaIn@SODPT NPs, a thicker encapsulating layer of around 6 nm (on average) was present (Figure 2a). The HAADF/EDX scan line analysis revealed the presence of Ga and In as the main elements along with those of O (Figure S6a), P, and S at lower concentrations (Figure S6b). This study was supported by X-ray photoelectron spectroscopy (XPS) analysis. The high-resolution XPS spectrum in the region of P 2p depicted in Figure 2b showed a peak at a binding energy of 135.2 eV, verifying the presence of P. Noticeably, the high-resolution XPS analysis for S 2p in Figure 2c showed a complex peak that could be deconvoluted in five peaks: the peak at 160.7 eV was assigned to Ga 3s which overlaps with S 2p region, two additional peaks at 161.9 and 163.0 eV assigned to S 2p_3/2_ and S 2p_1/2_, respectively, for Ga–S, and two other peaks at 165 and 166.1 eV corresponding to S 2p_3/2_ and S 2p_1/2_ for C–S–P. For comparison, XPS spectra were also acquired in the same region for bare EGaIn NPs and EGaIn@ODPA NPs displaying a single peak at around 160.4 eV associated with Ga 3s (Figure S7a,b). In addition, C 1s spectra (Figure S8) of EGaIn@ODPA and EGaIn@SODPT showed a peak at 284.8 eV assigned to −C–C– with higher intensity compared with that of the bare EGaIn, supporting the incorporation of the alkyl chain of the ligands. A low intense peak associated with C–C is also observed for EGaIn NPs (Figure S8a), which could be associated with adventitious carbon as it has been observed for EGaIn NPs synthesized in EtOH.^48^ For the O 1s, the peak at around 530 eV was attributed to the main contribution of Ga–O (from the oxide layer). For the modified NPs, additional peaks at higher binding energies were assigned to the presence of P–O, P=O, and P–O-metal (M) (Figure S8 and Table S1). Finally, the XPS spectra from Ga 2p and Ga 3d were very similar for all three samples (Figure S9). The successful functionalization was further corroborated by ATR FT-IR measurements (Figure S10 and Table S2). For both synthesized NPs, the peaks corresponding to C–H bonds are clearly present. In addition, a peak at 1082.4 cm^–1^ in the P=O and P–O regions of the spectra was observed for EGaIn@ODPA (Figure S10b), meanwhile for EGaIn@SODPT, the peaks appeared much broaden (Figure S10d). Peak position displacement or intensity changes were attributed to the interaction between the phosphonic acid group and the NP surface.^42,49^

**Figure 2:**
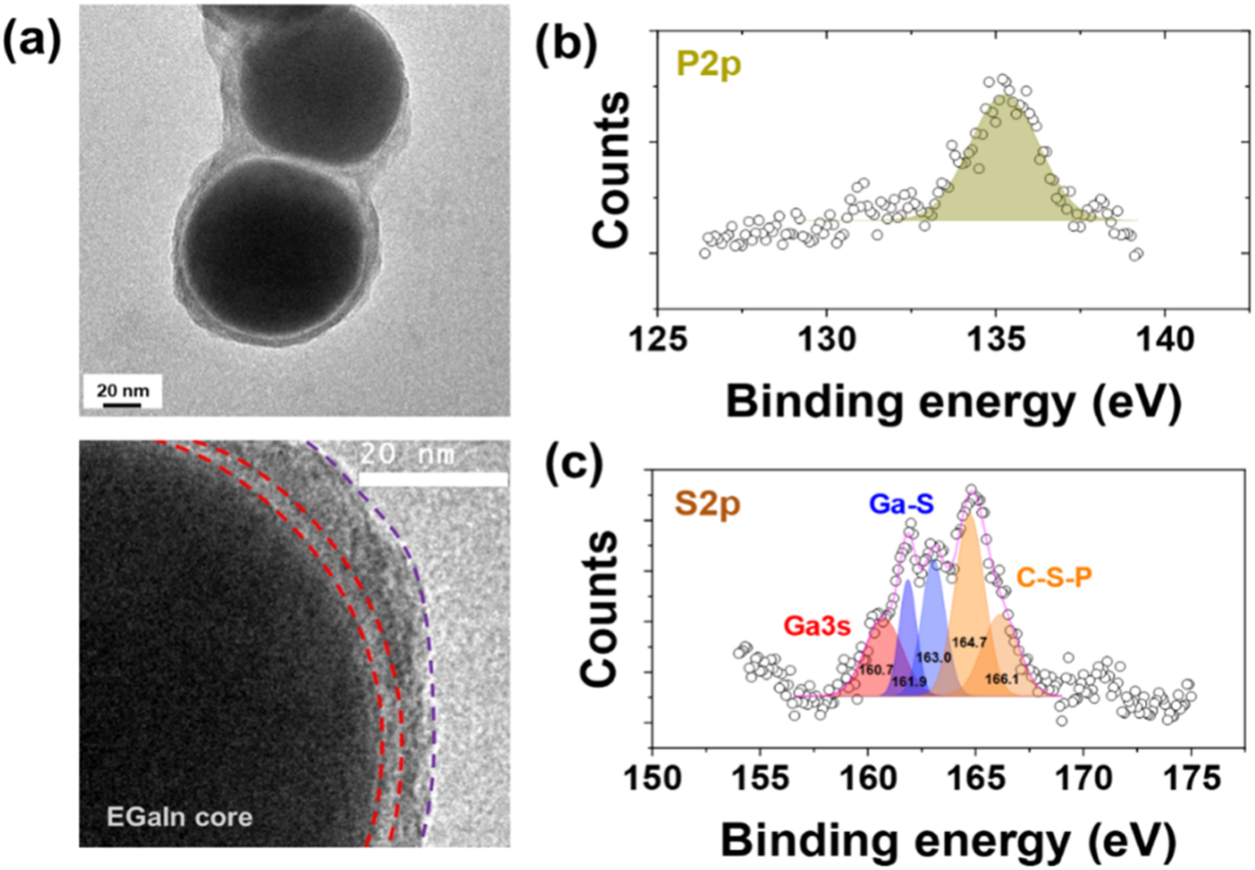
(a) (Top) HRTEM of EGaIn@SODPT NPs and (bottom) zoom-in of the NP/water interface with the oxide layer shown between red-dotted lines and the second hybrid shell border indicated in violet-dotted lines. High-resolution XPS spectra in the region of (b) P 2p and (c) S 2p.

The stability of the three systems was evaluated by DLS measurements. Figure 3 shows the correlograms of the different formulations over time. For the non-modified EGaIn NPs, after 7 days, the dispersion aggregated and could not be resuspended (Figure 3a). Instead, the presence of the ligand shell led to a clear stability improvement, allowing the colloidal suspensions to remain easily dispersible for up to 54 days. Nevertheless, in the case of EGaIn@ODPA NPs (Figure 3b), after 54 days, the presence of aggregates in the decay region of the correlogram was observed, indicating sample instability, which was not detected for EGaIn@SODPT NPs (Figure 3c). Additionally, the low variation in size distribution over time (Table S3) supported the potential of short-length phosphonic acid derivatives for stabilizing EGaIn NPs with a narrow size distribution in water. Further, the zeta-potential values displayed insignificant variation between day 1 and day 54 for both samples being from −58.7 ± 0.3 to −54.0 ± 3.3 mV (for EGaIn@ODPA) and from −61.0 ± 1.5 to −64.7 ± 1.5 mV (for EGaIn@SODPT). The long-term stability (up to 2 years) of an EGaIn@SODPT NP suspension stored at 4 °C was assessed using TEM and DLS. The results revealed that NPs retained their spherical shape with only a slight increase in size distribution (Figure S11). This enhanced stability is related to the different binding modes of the −S–PO­(OH)_2_ compared with −PO­(OH)_2_ discussed above, as well as promoted by the higher stability of the S–P bond upon hydrolysis.

**Figure 3:**
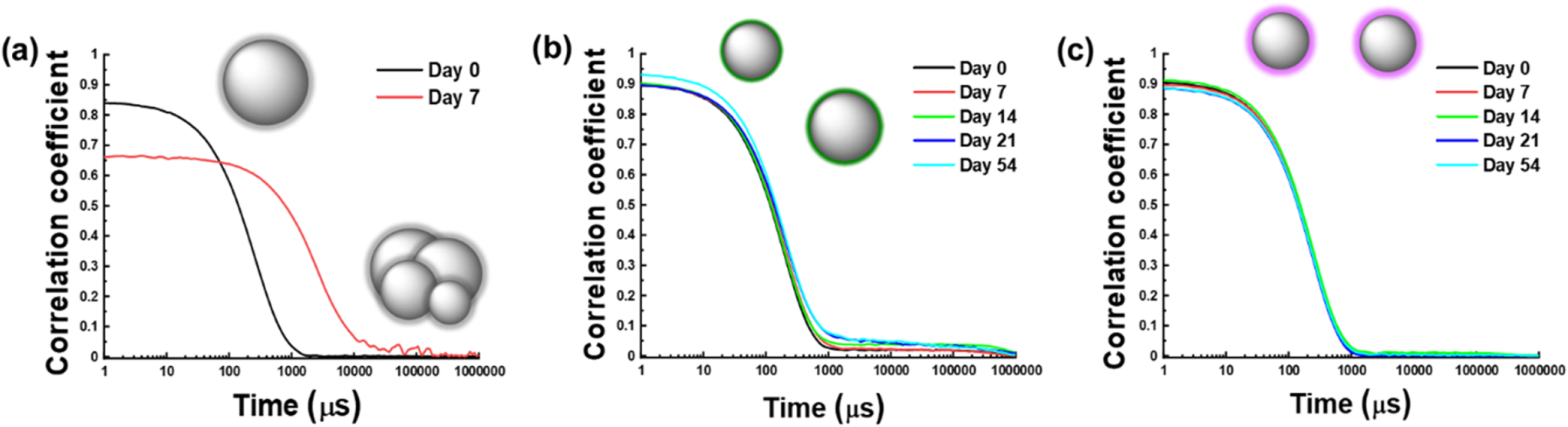
Correlograms obtained by DLS measurements of the synthesized EGaIn NPs: (a) non-modified EGaIn, (b) EGaIn@ODPA, and (c) EGaIn@SODPT.

Interestingly, the UV–vis spectra of the synthesized EGaIn NPs (Figure 4) showed that in the absence of ligand (Figure 4, top), a broad UV absorption band was observed, in agreement with the high polydispersity of the sample. On the contrary, in the case of the functionalized particles, a localized surface plasmon resonance (LSPR) peak^8,48^ was measured, with an absorption peak at 263 nm and a shoulder around 283 nm for EGaIn@ODPA NPs, and a well-defined peak at 263 nm for EGaIn@SODPT NPs. Additional experiments were performed to elucidate the effect of particle concentration and dispersion. On the one hand, UV–vis spectra were recorded for both concentrated and diluted EGaIn@ODPA NPs, as well as during the purification process of EGaIn@SODPT NPs via centrifugation. In both cases, a reduction in particle aggregation and polydispersity resulted in better-defined LSPR peaks (Figure S12). Furthermore, owing to the enhanced stability of EGaIn@SODPT NPs, a dialysis process was performed to isolate smaller nanoparticles. This led to a decrease in the average particle size from 154.6 ± 2.7 to 106.2 ± 1.0 nm, which correlated with a small red shift in the main LSPR peak, from 263 to 265 nm, and the emergence of a distinct shoulder at 285 nm, along with an additional, broader, and less intense peak around 325 nm (Figure S13). These results evidence that the optical response of the investigated LM particles clearly depends on different factors (i.e., interparticle coupling, NP concentration, size and dispersity, and Ga_2_O_3_/ligand shell thickness) which are inherently interdependent. We believe that the observation may contribute to broadening the application of plasmonic EGaIn NPs, particularly in aqueous media, which is relevant for biosensing events.

**Figure 4:**
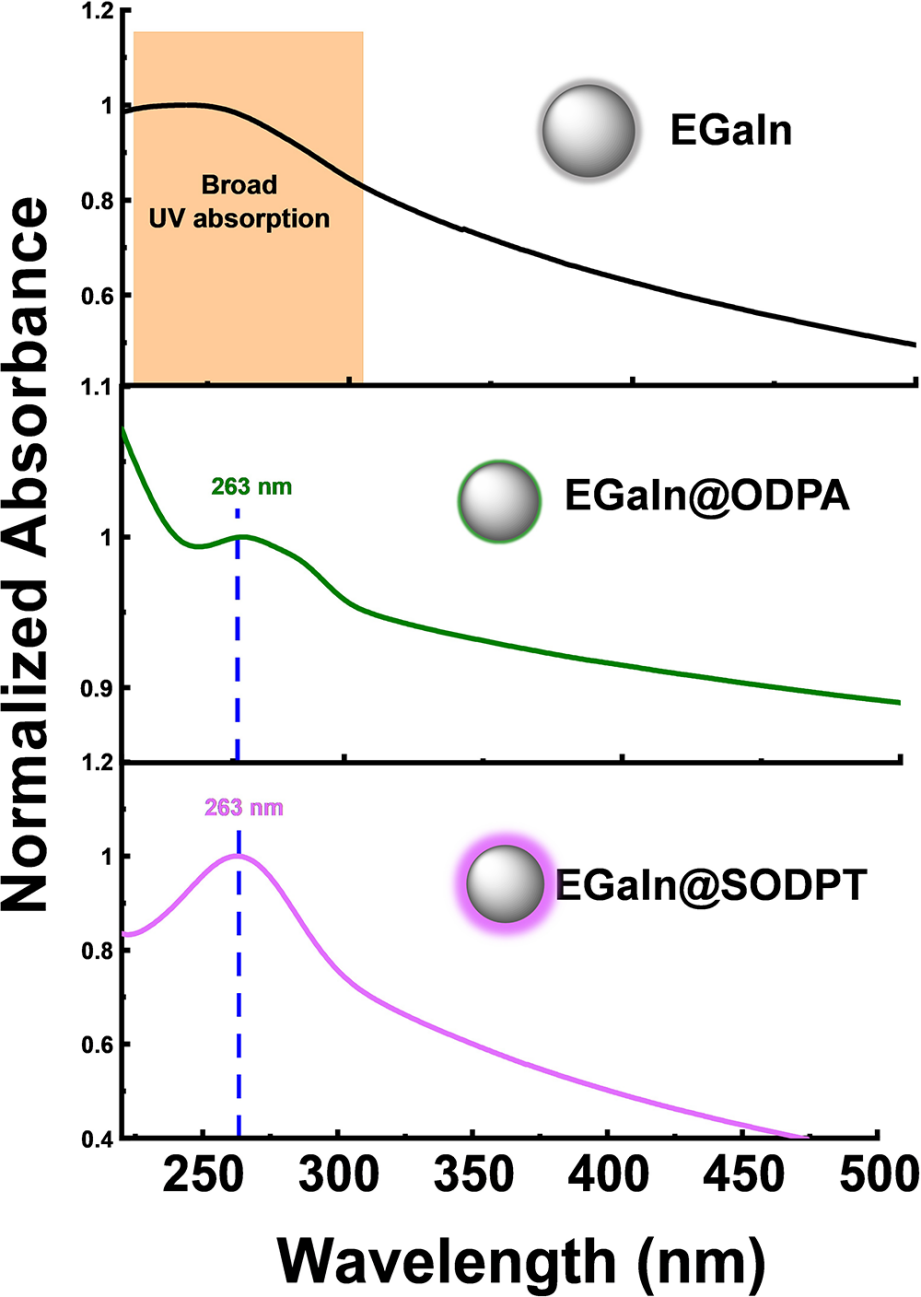
UV–vis absorption spectra of the colloidal suspension of EGaIn NPs in alkaline aqueous media. EGaIn without ligand (top), EGaIn@ODPA (middle), and EGaIn@SODPT (bottom) NPs.

Hence, the results presented above demonstrated that under the optimized synthetic conditions, both ligands allowed producing stable EGaIn NPs functionalized with small molecules. The use of the phosphorothioate derivative led to an enhanced colloidal suspension stability with a well-controlled size and morphology. This achievement motivated us to employ these sulfur-containing derivatives to conduct more complex surface reactions, with the goal of synthesizing functional EGaIn NPs, as discussed below.

### 3.2 Preparation of Redox-Active EGaIn NPs

To carry out the post-functionalization over EGaIn NPs, we used the well-known cross-linking conjugation reaction between an −NHS ester and a primary amine, here the 2,5-dioxopyrrolidin-1-yl-11-(phosphorothio) undecanoate (2,5-DPPNT) and the aminoferrocene (Fc-NH_2_) (Figure 5 and Scheme S1), respectively. The 2,5-DPPNT molecular structure contains a phosphorothioate anchoring group, an alkyl chain as a spacer and the −NHS ester as a terminal group (indicated in green in Figure 5).

**Figure 5:**
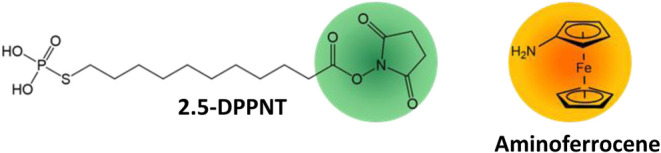
Molecular structure of the two components, ligand (2.5-DPPNT) and functional moiety (aminoferrocene), employed for the preparation of redox-active EGaIn NPs.

As the first synthetic approximation, the preparation of EGaIn@2.5-DPPNT NPs (Figure S14a) was attempted to later proceed with the reaction with Fc-NH_2_. EGaIn@2.5-DPPNT NPs obtained following the SODPT- and ODPA-optimized reaction conditions presented a hydrodynamic diameter of 152.9 ± 7.9 nm with a PDI value of 0.26 ± 0.04 and a zeta-potential value of −42.8 ± 1.76 mV. TEM analysis (Figure S14b) showed a thinner protecting shell compared with that of SODPT but with a similar rough morphology. However, the images also evidenced the presence of other byproducts generated during ultrasonication, which could not be removed during the purification process. The corresponding UV–vis spectrum showed a peak around 270 nm (Figure S14c), and the ATR-IR spectrum confirmed the molecule presence (Figure S14d). Unfortunately, the colloidal suspension showed poor stability over time (Figure S14e), as indicated by the correlograms of the initial sample and after 18 days with final particle sizes of 735.1 ± 633 nm. Such instability was attributed to the high reactivity of the −NHS ester group during ultrasonication considering that −NHS is prone to hydrolysis in alkaline environments. Thus, the planned subsequent reaction with the amino derivative was not conducted.

Alternatively, the method shown in Scheme S2 involved first synthesizing the phosphorothioate derivative bearing the Fc moiety through the reaction between 2.5DPPNT and Fc-NH_2_ in water (in 2:1 molar ratio (pH = 8)) under stirring in an ice bath for 30 min. The solution was directly used as the ligand without further purification. To promote ligand exchange reaction with SODPT ligands, a concentrated suspension of freshly prepared EGaIn@SODPT NPs was added to the freshly prepared ligand solution and the mixture was stirred for an additional 1 h at room temperature (see Experimental Section and SI for further details). Upon purification, the resulting particle size increased from 135 ± 4.8 d·nm (PDI 0.05 ± 0.02 and zeta potential = −62.4 ± 1.3 mV) to 246.6 ± 4.9 d·nm (PDI = 0.03 ± 0.01 and zeta potential = −69.6 ± 5 mV) (Figure S15a) being stable over 14 days (Figure S15d). TEM images (Figure S15b) corroborated the change of the NP hydrodynamic diameter with an LM core size of around 197 nm and a thick protecting layer with an average of 60 nm. HAADF/EDX line scan analysis was performed to determine the composition of this shell, showing the presence of Ga, O (from Ga_2_O_3_), and Fe over all NPs with S and P in lower amounts (Figure S15c). The formation of such a thick encapsulating shell could be driven by the supramolecular organization of the components involved in the NP synthesis. The most probable interactions taking place are the phosphonic acids with amino-based derivatives through hydrogen bonds^50–52^ and/or Fe­(III) salts and phosphonate moieties which have been reported to form organo-gels.^53^ To evaluate the redox activity of the obtained NPs, they were drop-cast on a glassy carbon electrode (GCE) disk acting as the working electrode. The cyclic voltammetry (CV) measurements revealed the presence of a well-defined reversible oxidation/reduction peak at *E* ^1/2^ = +0.04 V vs Ag/AgNO_3_ associated with the Fc/Fc^+^ redox process (Figure S15e) and the current intensity increased linearly with the scan rate from 50 mV/s to 1 V/s (Figure S15e), which is indicative of surface-confined redox species. Thus, while ligand exchange could not be proven and the reaction mechanism remained uncertain, the incorporation of the ferrocene moiety was confirmed.

To gain further insight into the reactivity of EGaIn@SODPT NPs with the two ligands used in this study, NPs were incubated separately with either 2.5-DPPNT or Fc-NH_2_ only. In the first case, the reaction led to NPs with a size distribution of 149.9 ± 1.0 d·nm (PDI = 0.11 ± 0.02; zeta potential = −45.3 ± 0.5 mV) and, in the case of Fc-NH_2_, the NP size increased up to 325.4 ± 6.9 d·nm (PDI 0.17 ± 0.02; zeta potential of −53.5 ± 1.0 mV) (Figure S16a), and with slight size and zeta-potential variations up to day 14 (Figure S16b,c). TEM images of EGaIn@SODPT/Fc-NH_2_ revealed a completely different morphology showing a yolk–shell structure (Figure S16e). Interestingly, HAADF/EDX analysis indicated a displacement of Ga toward the outer shell of NPs, leading to a metallic core composed mainly of In (Figure 6). These particles showed electrochemical activity displaying a peak at *E* ^1/2^ = +0.098 V (Δ*E* = 98 mV at 0.1 V/s, vs Ag/AgNO_3_) in CV, which was not observed for EGaIn@SODPT/2.5DPPNT NPs (Figure S17). Based on the standard redox potentials of Ga^3+^ (*E* ^0^ (Ga^3+/^Ga) = −0.53 V vs SHE and *E* ^0^ (Fe^2+/Fe3+^) = 0.4 V vs SHE), the obtained results suggest that a galvanic replacement reaction might be taking place. Hence, this is a simple novel approach to modify the composition of EGaIn-based NPs.

**Figure 6:**
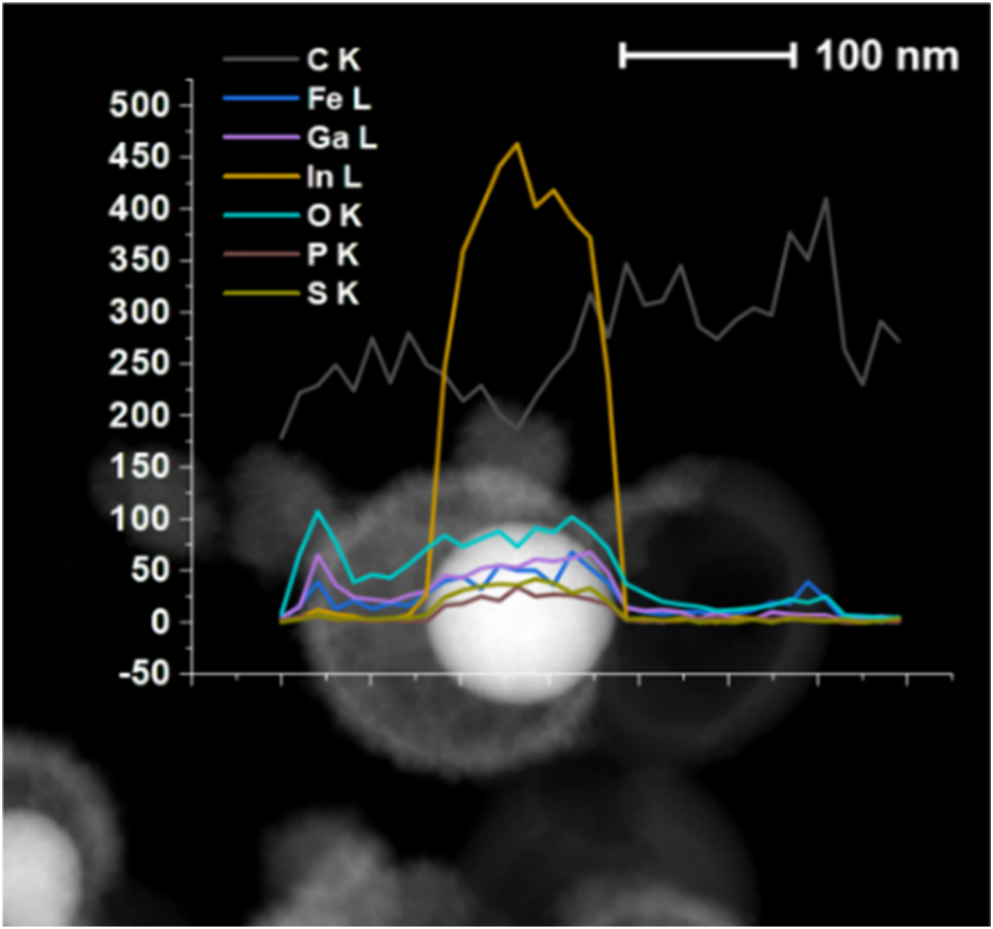
HAADF/EDX chemical scan line analysis for EGaIn@SODPT NPs after incubation with Fc-NH_2_.

Finally, with the aim to synthetically simplify even more the incorporation of the Fc moiety, a one-pot strategy was designed (Figure 7a and Scheme S3). For that, freshly prepared EGaIn@SODPT NPs were combined with a solution containing both ligands in a 1:1 molar ratio (2.5DPPNT:Fc-NH_2_), stirred for 1 h at room temperature, and finally purified by centrifugation. From the TEM images, a coating shell with a thickness of around 23 nm was observed (Figure 7b) and by HAADF/EDX line scan analysis, the presence of Ga and In as major components was confirmed, as well as Fe distributed around NPs, along with other elements in lower amounts (Figures 7c and S18). The resulting particles showed a size distribution of 162.8 ± 1.8 d·nm (Figure 7d; PDI = 0.031 ± 0.029; zeta potential = −58.2 ± 1.56 mV) stable up to 34 days, as seen by DLS (Figure S19). The UV–vis spectrum of the resulting NPs showed a main peak around 281 nm (18 nm red-shifted compared with EGaIn@SODPT NPs) and the contribution of Fc at the 310–370 nm region (Figure 7e). The electrochemical characterization performed by CV confirmed the presence of a reversible oxidation/reduction peak from the Fc/Fc^+^ pair (Figure 7f), at *E* ^1/2^ = 0.03 V vs Ag/AgNO_3_ and a linear increase of the current intensity versus the scan rate (Figure S20). Thus, although the exact nature of the NP/ligand interactions, whether covalent or non-covalent, and the ligand organization at the NP surface have yet to be elucidated, this strategy enabled the reproducible synthesis of EGaIn NPs with electrochemical activity imparted by the incorporation of Fc moieties. The formation of the thicker outer layer in EGaIn@SODPT-2.5DPPNT/Fc NPs can be justified through a combination of supramolecular assembly processes and ligand reorganization, as evidenced by the experiment. The observed ∼23 nm shell in the one-pot synthesis (and ∼78 nm in the stepwise approach) highlights the critical role of non-covalent forces and ligand dynamics.

**Figure 7:**
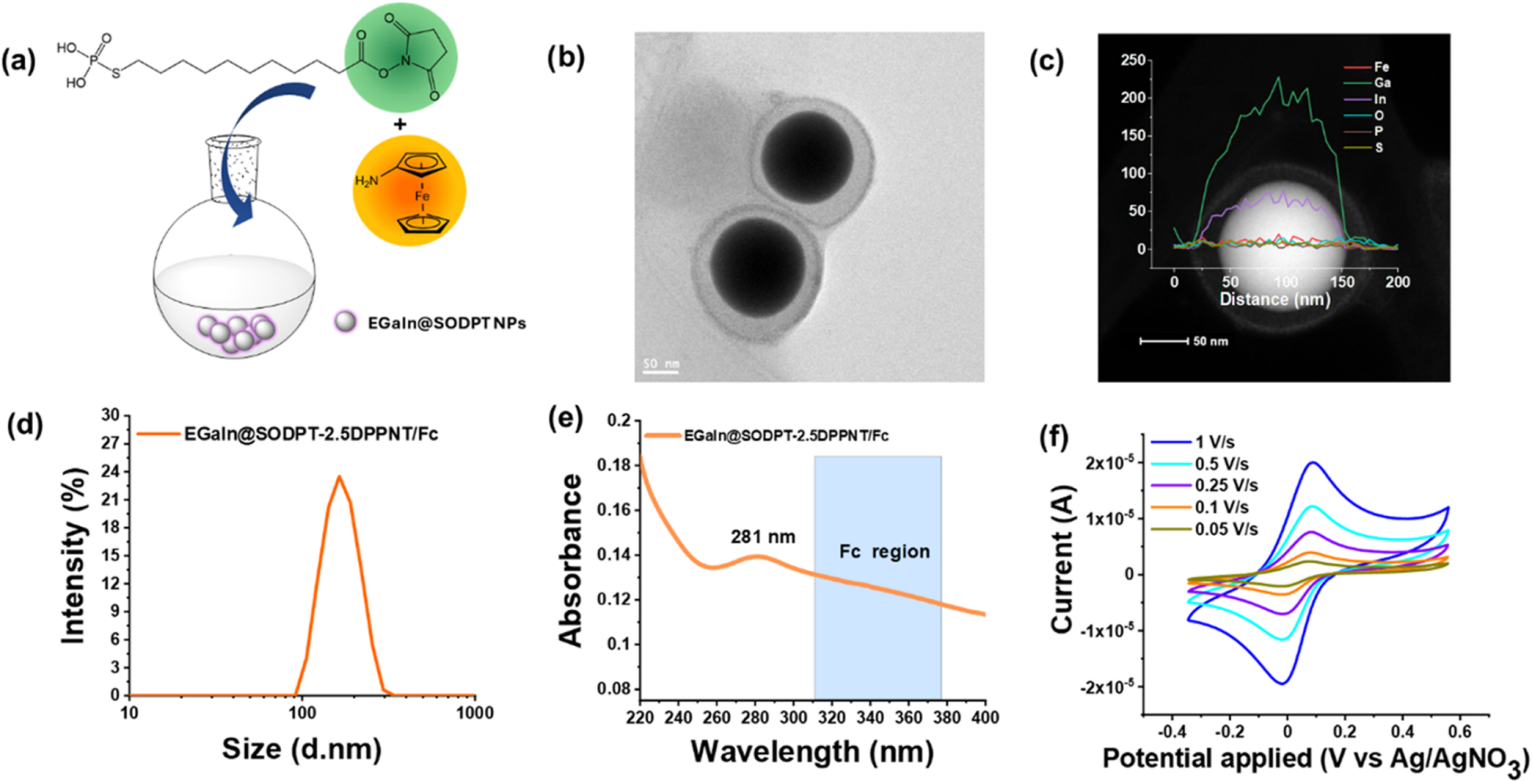
(a) Scheme of the one-pot surface modification to obtain EGaIn@SODPT-2.5DPPNT/Fc, (b) TEM image (scale 50 nm), (c) HAADF/EDX chemical line scan analysis, (d) size distribution of EGaIn@SODPT-2.5DPPNT/Fc NPs, (e) UV–vis spectrum, and (f) cyclic voltammetry (CV) at different scan rates in V/s.

## 4 Conclusions

The findings presented in this work contribute to the advancement toward sustainable and highly reproducible synthetic strategies to obtain EGaIn NPs with well-defined size and morphology. The successful functionalization of EGaIn NPs with low molecular weight derivatives featuring phosphonic acid as an anchoring group for Ga_2_O_3_ in aqueous solution has been demonstrated. Compared with thiols that are commonly employed to functionalize LMPs through Ga–S bonds, phosphonic acids form strong, stable P–O–Ga bonds with the oxide layer of EGaIn particles, and the introduction of phosphorothioates enables dual coordination via both phosphonate oxygen atoms to oxidize Ga and S atoms to metallic Ga exposed during ultrasonication. This dual-binding capacity results in a robust ligand shell, which we demonstrate leads to superiorcolloidal stability (up to two years). Furthermore, this approach avoids the use of polymers, which create a thick protective layer that may limit their application, for instance, in electronics.

By examining the impact of different pH conditions, the study revealed that alkaline environments afford enhanced control over NP size, distribution, and stability, especially for EGaIn@SODPT. Remarkably, EGaIn NPs with electrochemical activity were effectively produced, achieved by incorporating Fc moieties into the ligand shell. Notably, the reaction between the synthesized EGaIn NPs and Fc-NH_2_ induced a phase transition that modified the physicochemical properties of EGaIn NPs. In summary, we believe that this work not only contributes to a deeper understanding of the synthesis of stable EGaIn NPs in environmentally friendly conditions but also explores strategies for developing functional liquid metal NPs. NPs developed herein hold potential for applications such as electrocatalysis, where the anchored electrochemically active unit could act as a redox mediator working in synergy with the catalytic activity of the liquid metal.

## Supplementary Material



## Notes

Spectroscopic characterization of the synthesized EGaIn NPs (PDF)
