# Supplementary material for: Surface Modification and Stabilization of Eutectic Gallium Indium Nanoparticles with an Electrochemically Active Ligand Using Low Molecular Weight Phosphorothioates in Water
Source: ACS Omega. 2025 Jun 11;10(24):25894–903. doi: 10.1021/acsomega.5c02237 (PMC12199008; doi:10.1021/acsomega.5c02237)
Supplement: Supplementary file 1 [file ao5c02237_si_001.pdf]

Supporting information

Surface Modification and stabilization of Eutectic Gallium Indium Nanoparticles with an Electrochemically Active Ligand using Low Molecular Weight Phosphorothioates in Water

José Catalán-Toledo, Jordi Romero-Pallejà and Núria Crivillers

Institut de Ciència de Materials de Barcelona (ICMAB-CSIC), Campus de la UAB s/n, Bellaterra, 081093, Spain.

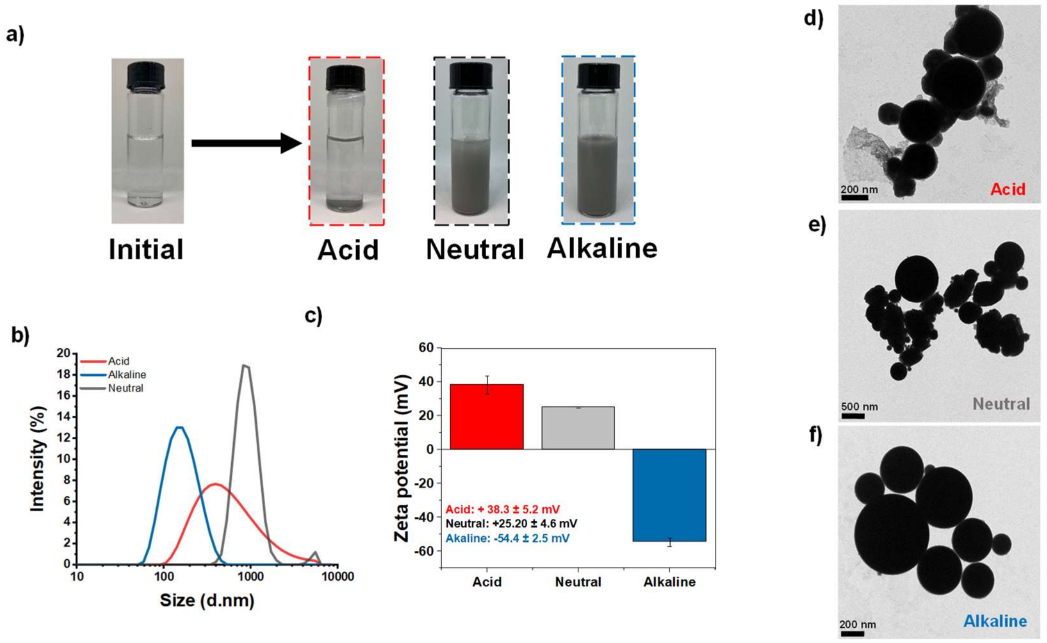

Figure S1. a) Pictures of vials containing bulk EGaIn metal (left) and the resulting suspensions after ultrasonication in different pHs (right). b) Hydrodynamic diameter distribution of the EGaIn NPs at different pH conditions measured by DLS. c) Zeta potential (Inset: values), error bars represent standard deviation. d-f) TEM images of non-functionalized EGaIn NPs prepared from acid (scale: 200 nm), neutral (scale: 0.5 µm) alkaline (scale: 200 nm) aqueous solutions.

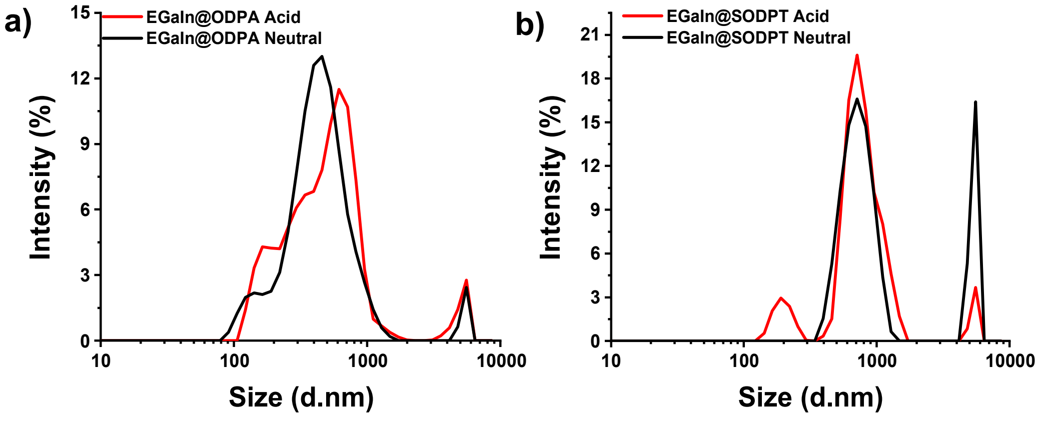

Figure S2. Hydrodynamic diameter distribution in acid and neutral pHs for a) EGaIn@ODPA and b) EGaIn@SODPT NPs.

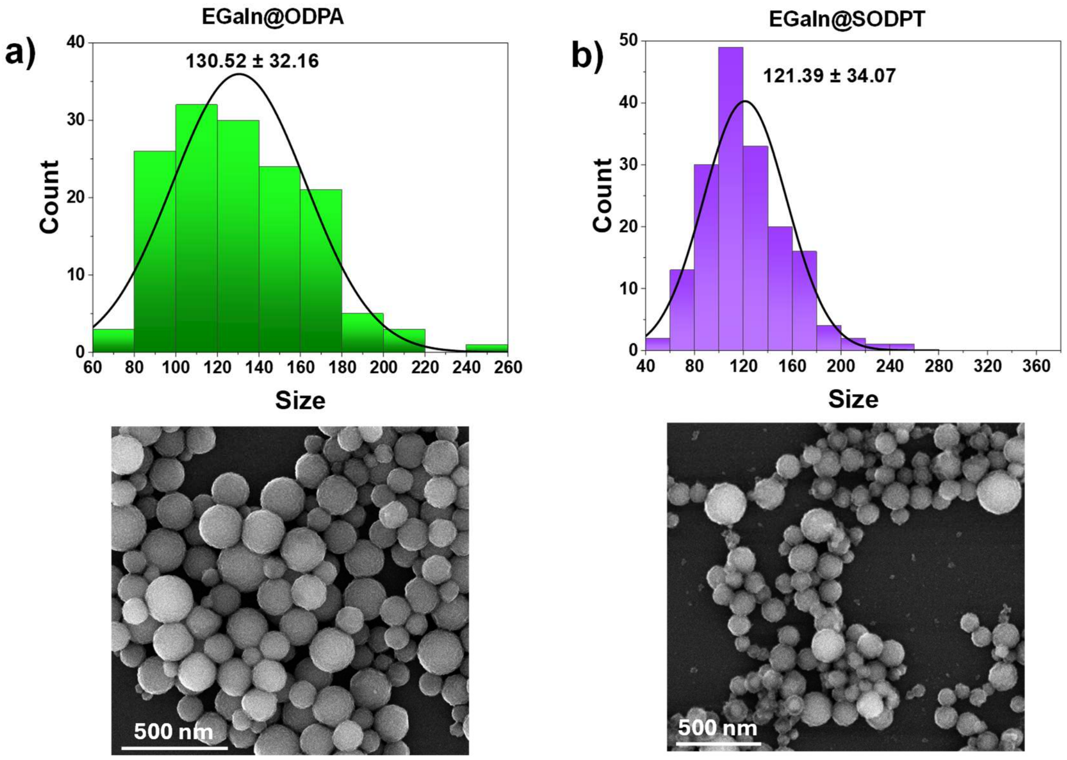

Figure S3. STEM images and size distribution histograms of a) EGaIn@ODPA and b) EGaIn@SODPT NPs.

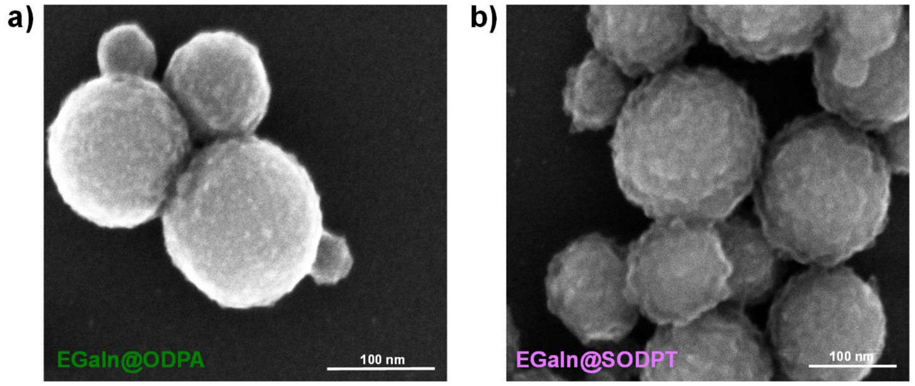

Figure S4. SEM images of a) EGaIn@ODPA and b) EGaIn@SODPT NPs.

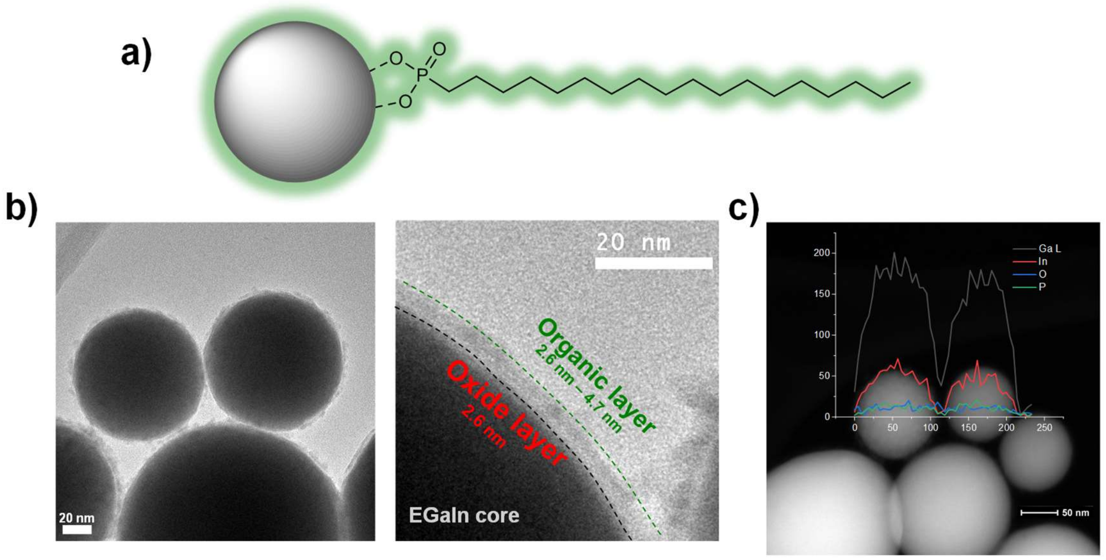

Figure S5. a) Scheme of EGaIn@ODPA NPs. b) and c) HRTEM images showing the different layers covering the LM core. Inset: HAADF / EDX scan line chemical analysis, indicating the most abundant elements.

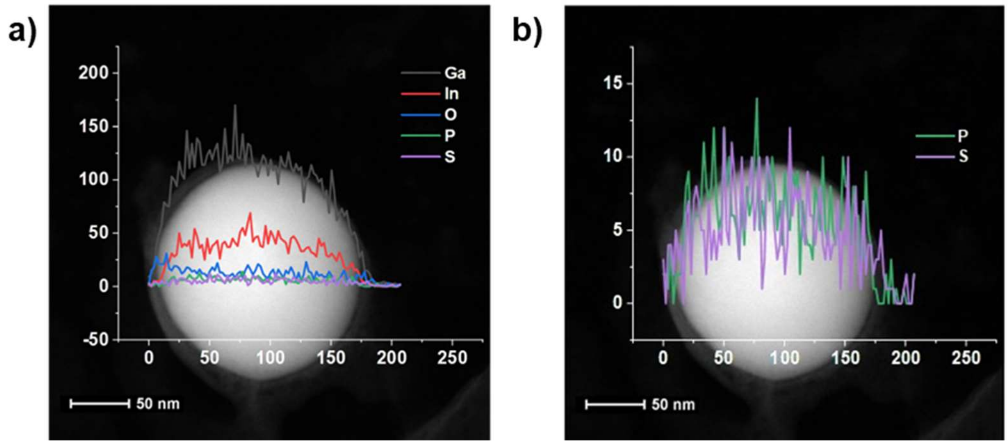

Figure S6. a) HRTEM image and HAADF / EDX scan line chemical analysis, indicating the most abundant elements of EGaIn@SODPT NPs and b) scan line for only P and S for clarity.

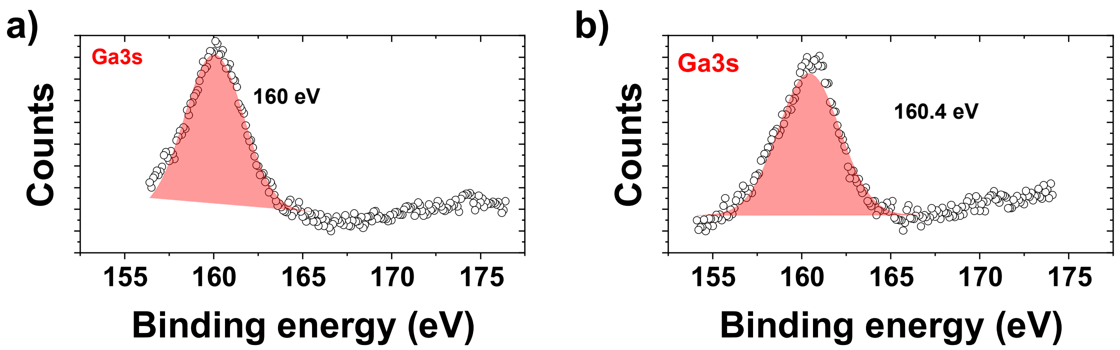

Figure S7. High-resolution XPS spectra of Ga3s region of a) bare EGaIn and b) EGaIn@ODPA NPs.

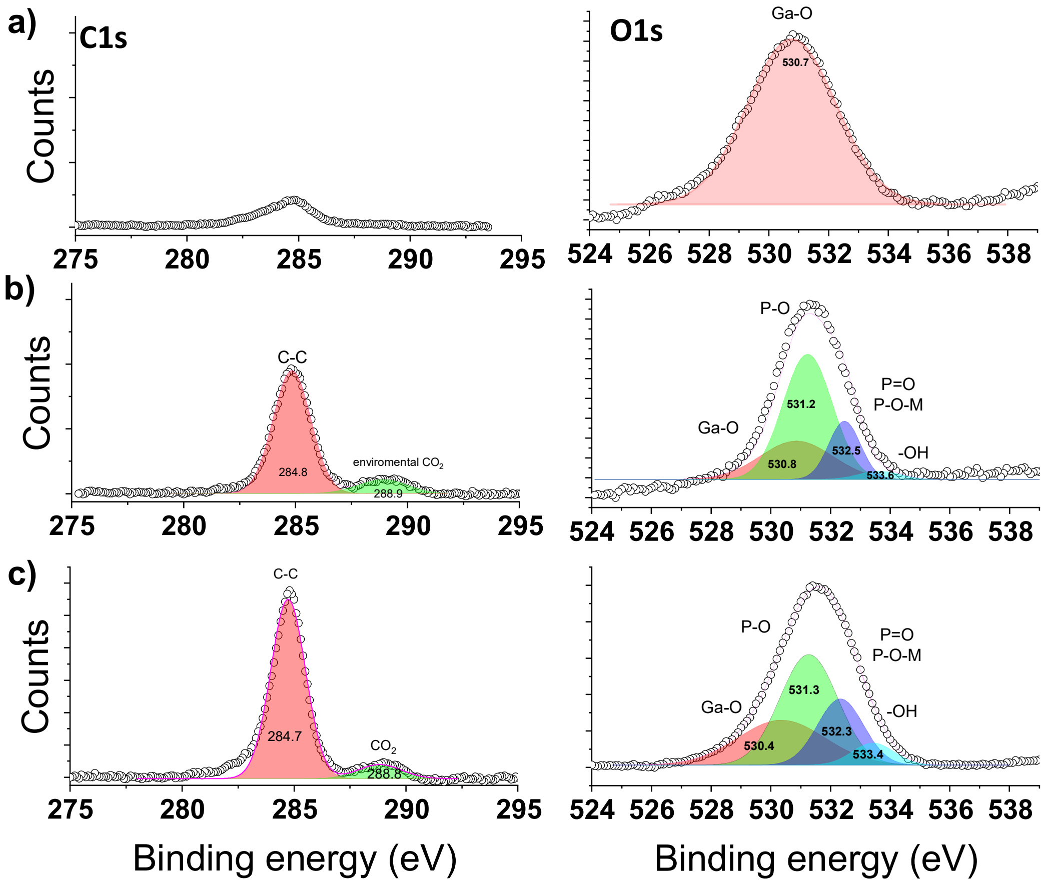

Figure S8. High-resolution XPS spectra for C1s and O1s region of a) bare EGaIn, b) EGaIn@ODPA and c) EGaIn@SODPT NPs.

Table S1. XPS peaks binding energy of O1s for the synthesized EGaIn NPs.

Interaction	Bare EGaIn	EGaIn@ODPA	EGaIn@SODPT
Ga-O	530.7 eV	530.8 eV	530.4 eV
P-O	-	531.2 eV	531.3 eV
P=O / P-O-M	-	532.5 eV	532.3 eV
-OH	-	533.6 eV	533.4 eV

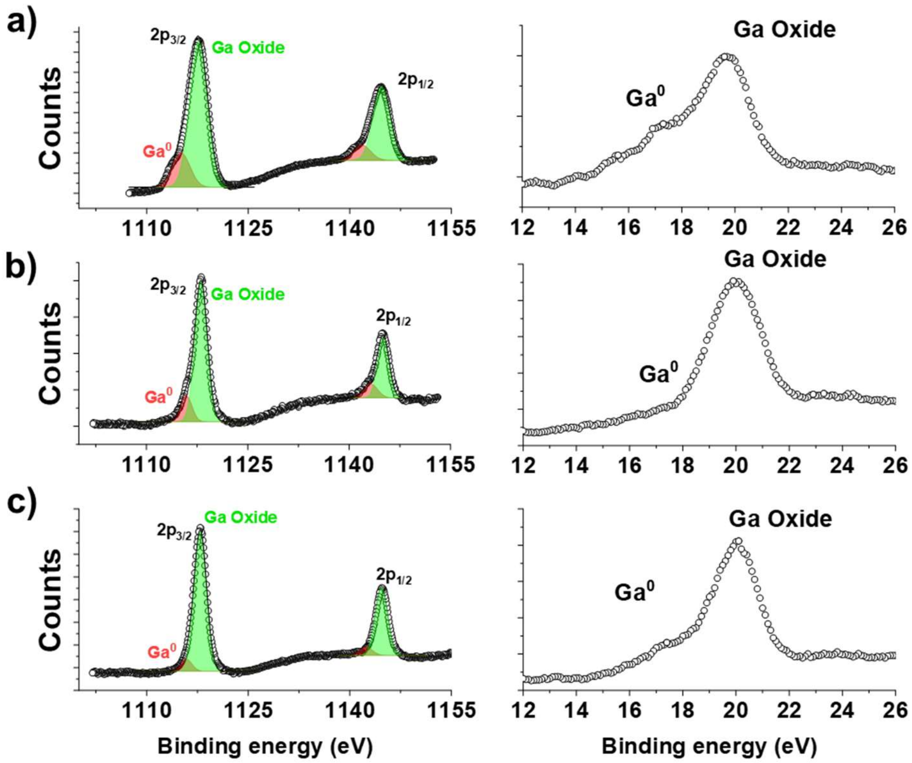

Figure S9. XPS high-resolution spectra for Ga2p (left) and Ga3d (right) region of a) bare EGaIn, b) EGaIn@ODPA and c) EGaIn@SODPT NPs.

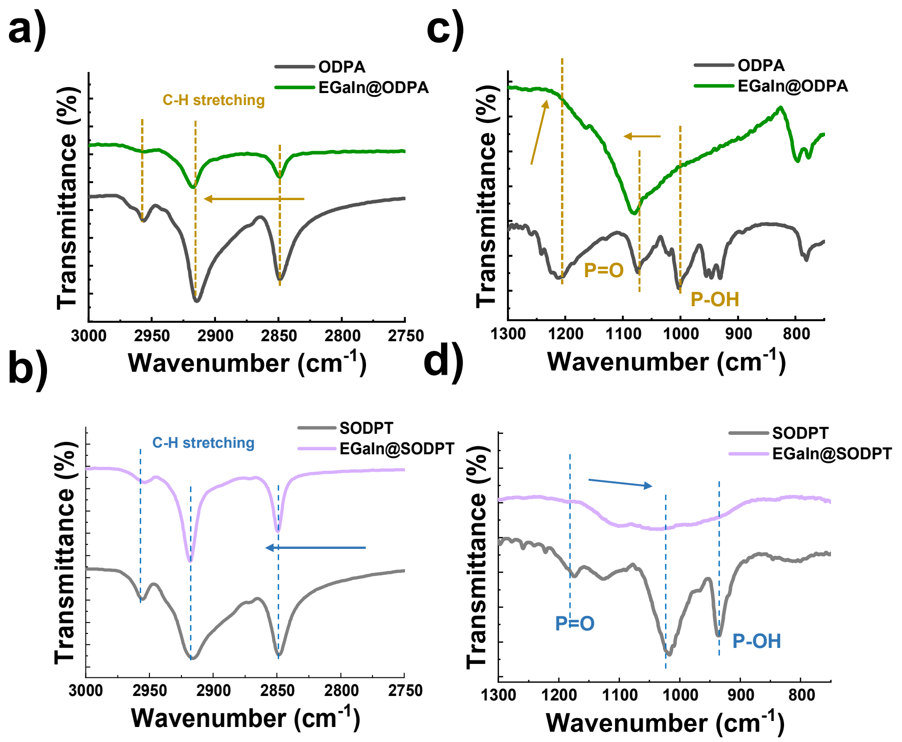

Figure S10. ATR-IR spectra of EGaIn functionalized nanoparticles and respective pure ligands for a-c) EGaIn@ODPA and b-d) EGaIn@SODPT NPs.

Table S2. Main IR peaks of the ligands and of the synthesized EGaIn@PA NPs.

	Wavenumber (cm^{-1})			
**Chemical group**	**ODPA**	**EGaIn@ODPA**	**SODPT**	**EGaIn@SODPT**
**C-H Stretching**	2848.3	2849.8	2848.4	2848.8
	2914.4	2917.3	2915.9	2918.3
	2955	-	2955.8	2954.4
**P=O Stretching**	1212.5	-	1174.3	-
	-		1128.6	-
**P-O Stretching**	1074.2	1082.4	1018.2	1106.0
	1002.8		-	
	936.0			

Table S3. Evolution of the hydrodynamic diameter mean value (d), PDI and zeta potential values for the synthesized EGaIn NPs modified with phosphonic acid derivatives.

	EGaIn@ODPA			EGaIn@SODPT		
**Day**	**d (nm)**	**PDI**	**z-pot (mV)**	**d (nm)**	**PDI**	**z-pot (mV)**
1	127.9 ± 2.2	0.097 ± 0.016	-58.7± 0.3	154.3 ± 4.1	0.036 ± 0.009	-61.0 ± 1.7
7	134.6 ± 0.9	0.159 ± 0.050	-58.2 ± 0.2	162.9 ± 4.7	0.068 ± 0.023	-60.6 ± 2.2
14	126.8 ± 2.7	0.091 ± 0.010	-53.0 ± 1.2	161.9 ± 6.5	0.082 ± 0.025	-57.5 ± 0.7
21	138.4 ± 5.0	0.243 ± 0.041	-57.1 ± 0.6	156. 1± 6.8	0.020 ± 0.023	-58.5 ± 1-3
54	140.2 ± 2.3	0.217 ± 0.085	-54.0 ± 3.3	160.6 ± 4.0	0.041 ± 0.006	-64.7 ± 1.5

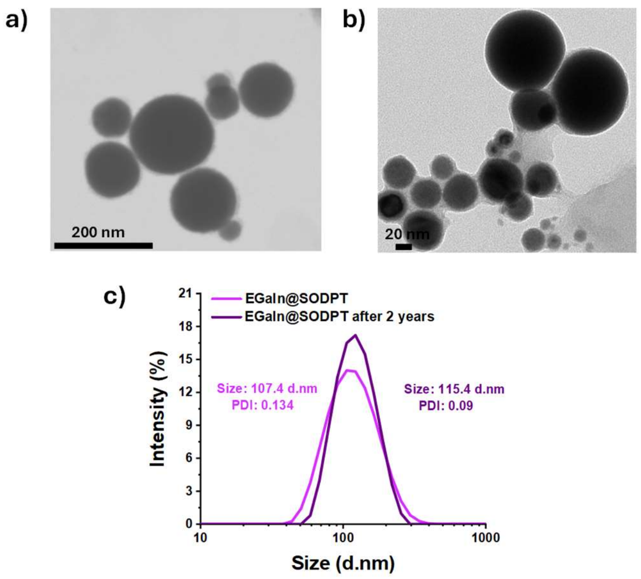

Figure S11. TEM images of EGaIn@SODPT NPs a) freshly prepared and b) after 2 years stored at 4ºC, c) size distribution measured by DLS, before and after the long-term storage.

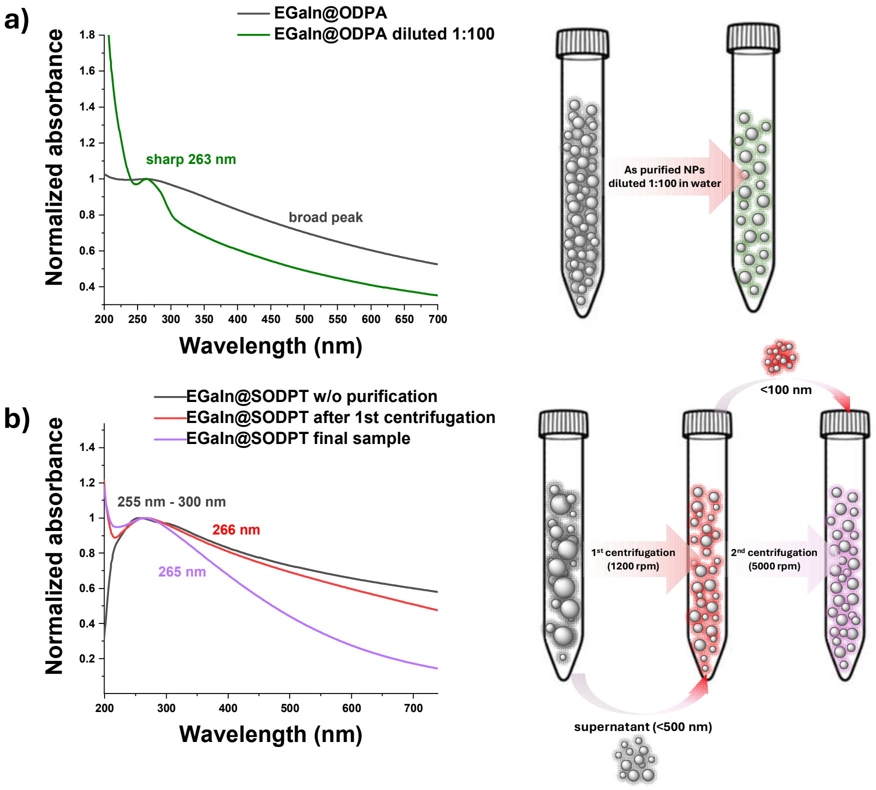

Figure S12. a) Optical characterization of EGaIn@ODPA NPs at different concentration. The UV-Vis peak at ~263 nm broadens with increasing concentration due to interparticle coupling and scattering effects; b) UV-Vis spectra of EGaIn@SODPT NPs during purification. A clearer UV plasmon peak emerges after the second centrifugation step, indicating removal of larger or aggregated particles.

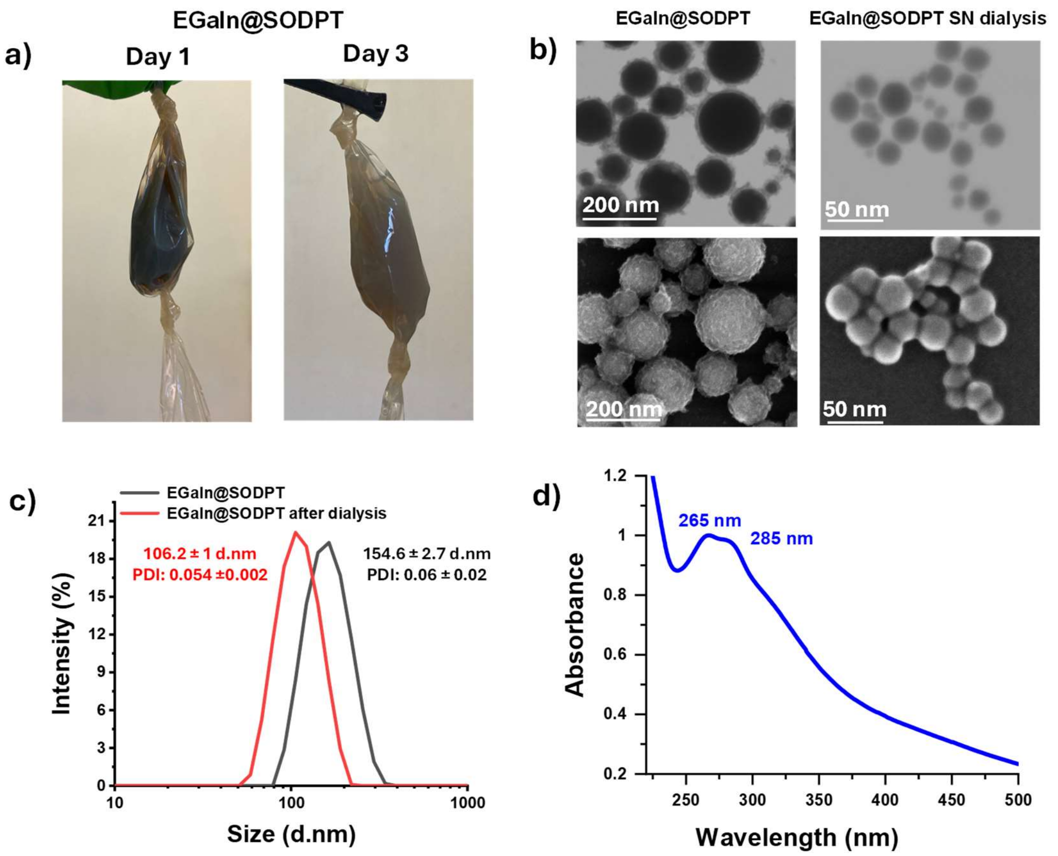

Figure S13. a) Dialysis of freshly prepared EGaIn@SODPT NPs after 3 days. b) STEM of EGaIn@SODPT before and after dialysis. c) Size distribution of EGaIn@SODPT NPs before and after dialysis and d) UV-Vis of dialyzed EGaIn@SODPT.

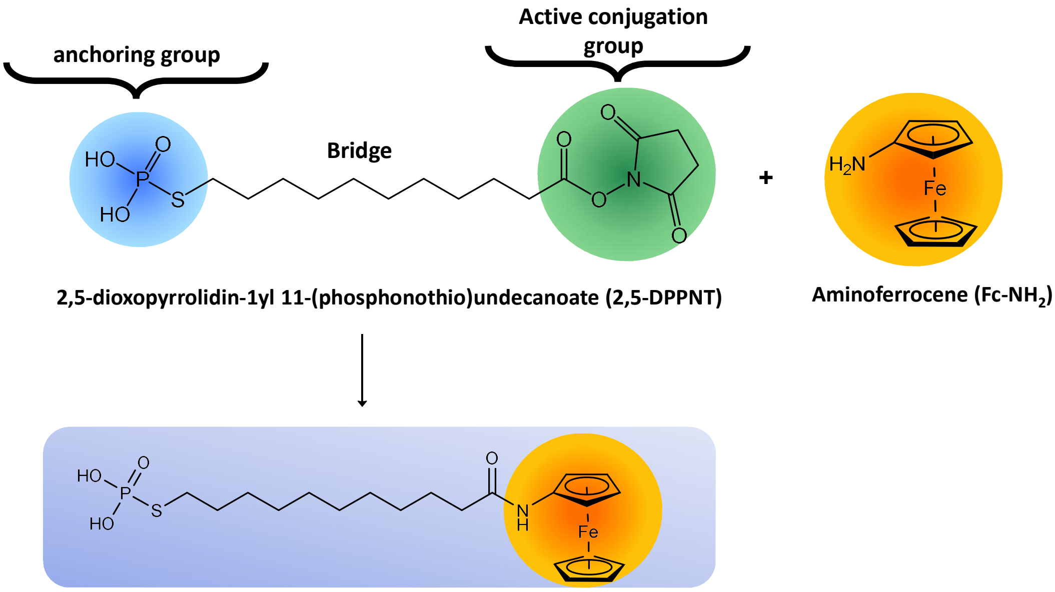

Scheme S1. Molecular structure of 2.5DPPNT and amino ferrocene ligands and scheme of the crosslinking conjugation reaction.

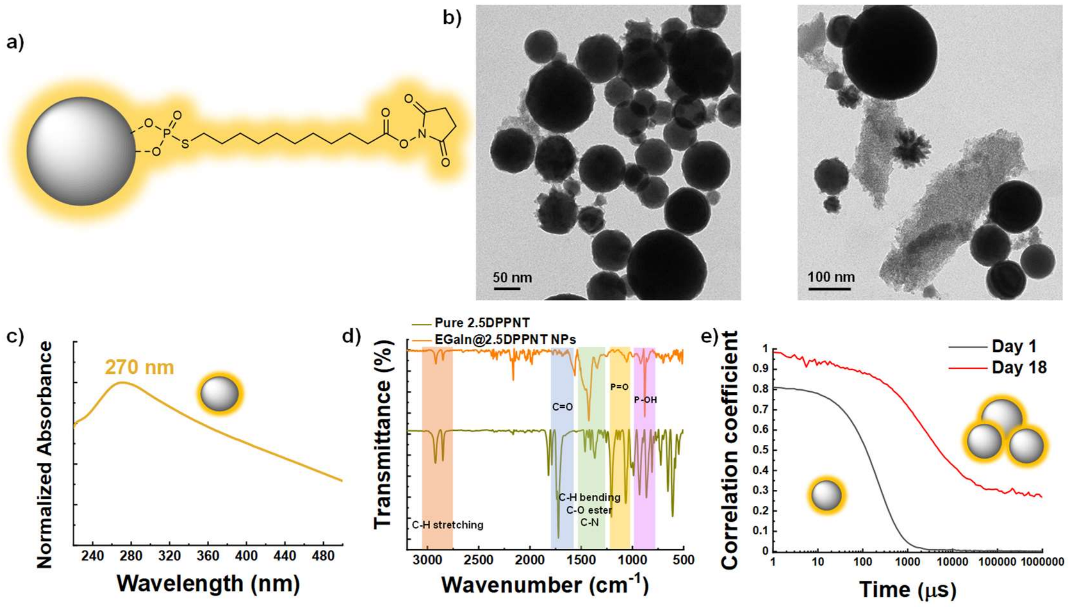

Figure S14. EGaIn@2.5DPPNT NPs characterization**.** a) Scheme of EGaIn@2.5DPPNT NPs. b) TEM images from two different regions of the same sample, a region with main presence of NPs (left) and another region showing reaction by-products of the NPs synthesis (right). c) UV-vis spectra, d) ATR-IR spectra of pure ligand (green) and synthesized NPs (orange). e) Correlograms corresponding to day 1 and day 18, showing low stability.

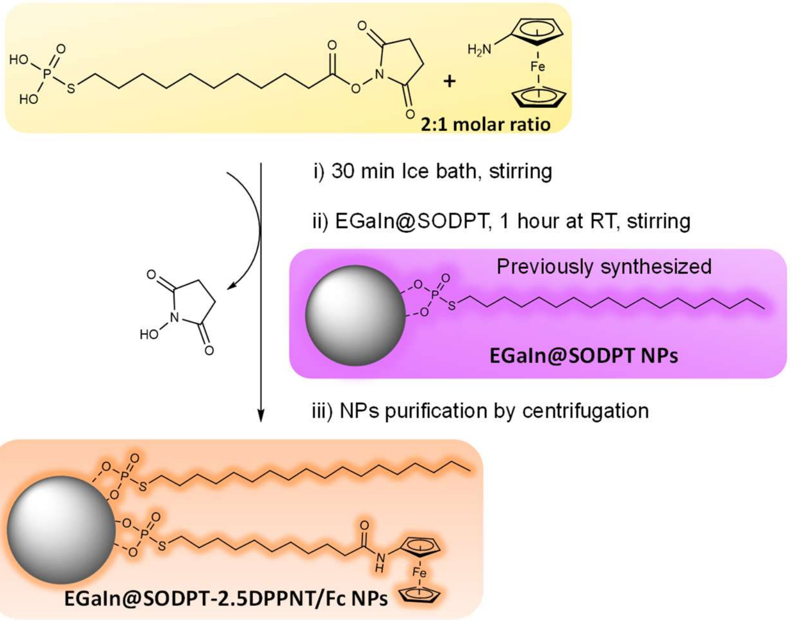

Scheme S2. Scheme of the post-functionalization strategy for EGaIn NPs. i) A 2.5DPPNT and Fc-NH_2 aqueous solution in a 2:1 molar was stirred for 30 min in an ice bath. ii) The freshly prepared EGaIn@SODPT NPs were poured into the reaction mixture, and the mixture was let to stir for 1 hour at RT. iii) NPs were purified following the stablished method of purification by centrifugation.

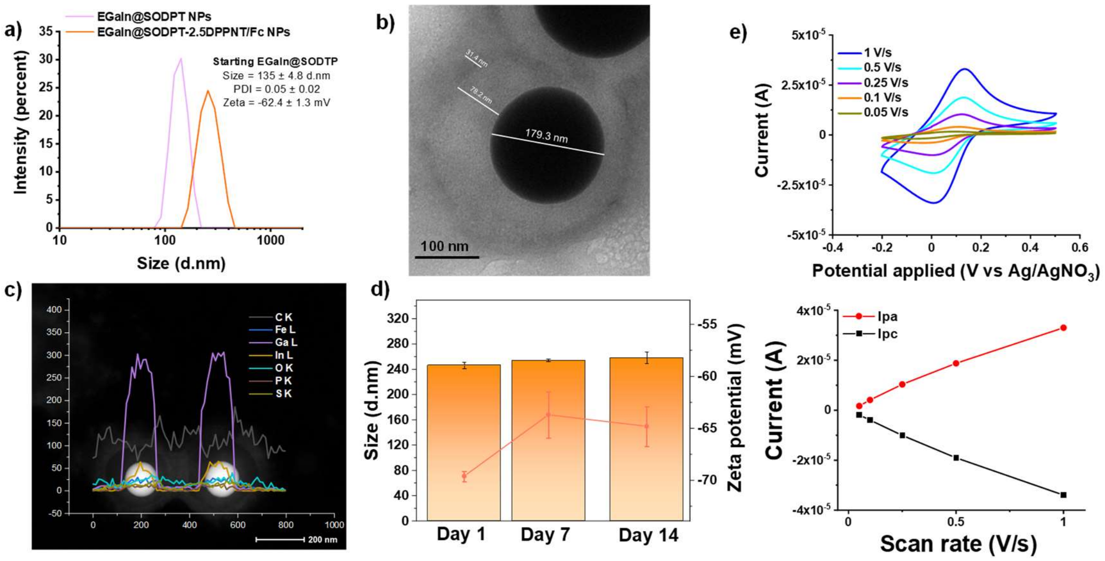

Figure S15. Characterization of the NPs obtained following the reaction depicted in Scheme 2. a) Hydrodynamic size distribution for freshly prepared EGaIn@SODPT NPs and after functionalization (Inset: EGaIn@SODPT NPs data). b) HRTEM image of functionalized sample, indicating the thickness of the different layers. c) HAADF-EDX chemical line scan analysis of the functionalized NPs. d) Hydrodynamic diameter and zeta potential up to 14 days. e) Cyclic voltammetry of the synthesised NPs at different scan rates (top) and plot of the I_{pa} and I_{pc} as a function of the scan rate (bottom) (0.1 M $TBAFP_6$ in CH_2CN. WE:GCE, RE: Ag/$AgNO_3$ and CE: Pt wire).

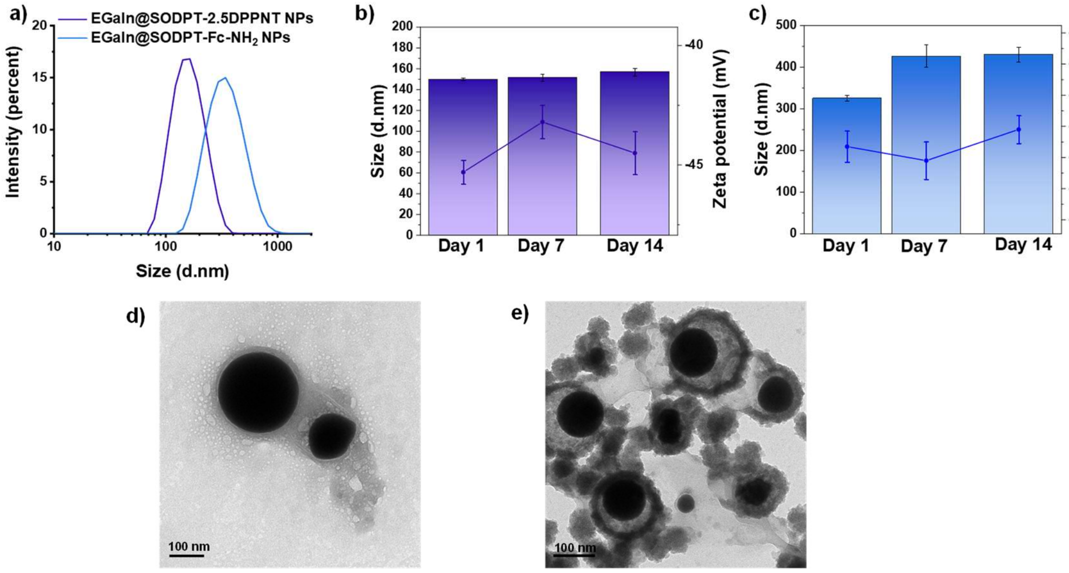

Figure S16. a) Hydrodynamic diameter distribution of EGaIn@SODPT NPs after incubation with 2.5DPPNT (purple) or Fc-NH_2 (blue). Stability up to 14 days indicating size and zeta potential for b) EGaIn@SODPT-2.5DPPNT NPs and c) EGaIn@SODPT-Fc-NH_2 NPs. d-e) TEM images for EGaIn@SODPT-2.5DPPNT and EGaIn@SODPT-Fc-NH_2 NPs, respectively.

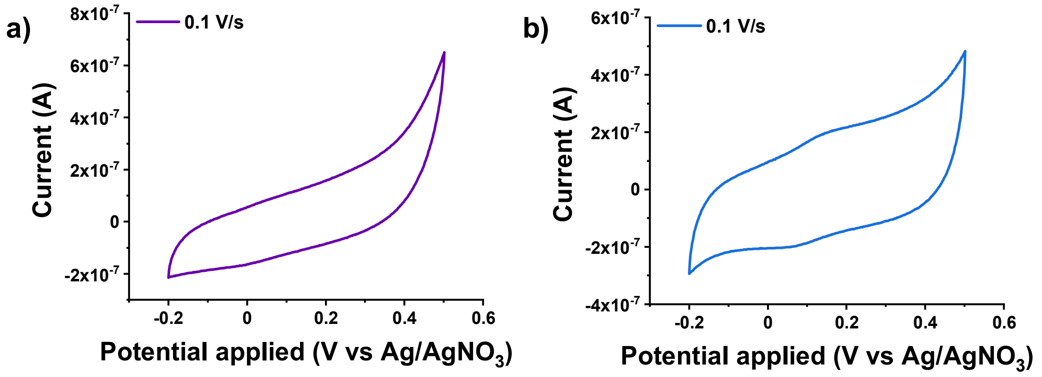

Figure S17. Cyclic voltammetry of EGaIn@SODPT-2.5DPPNT and EGaIn@SODPT-Fc-NH_2 NPs. (0.1 M $TBAFP_6$ in CH_2CN. WE: GCE, RE: Ag/$AgNO_3$ and CE: Pt wire).

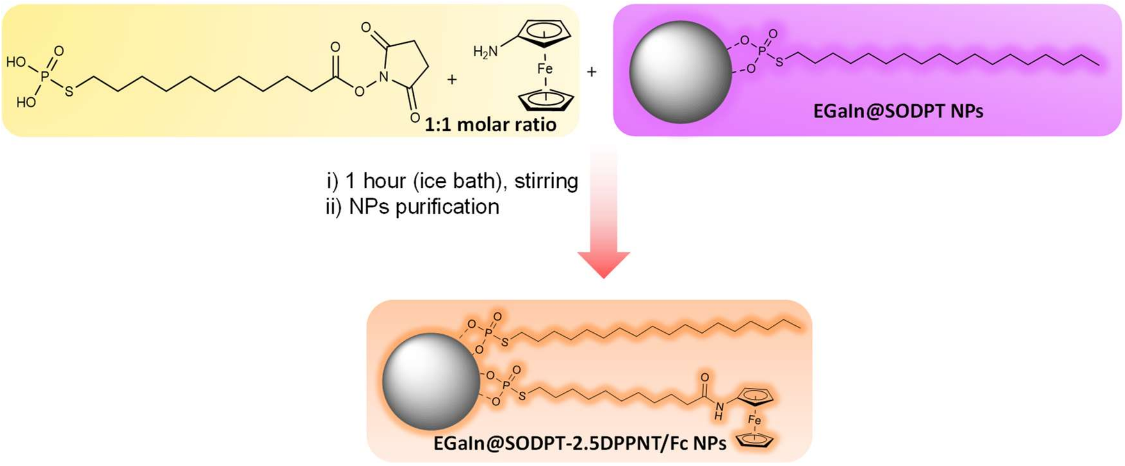

Scheme S3. Scheme of the one-pot post-functionalization strategy of EGaIn@SODPT NPs. i) An aqueous solution of 2.5DPPNT and Fc-NH_2 in a 1:1 molar and freshly prepared EGaIn@SODPT NPs were mixed and stirred for 1h at RT. ii) NPs were purified following the stablished method of purification by centrifugation.

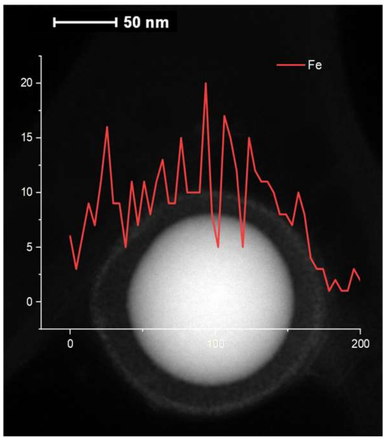

Figure S18. HAADF/EDX of Fe in EGaIn@SODPT-2.5DPPNT/Fc NPs.

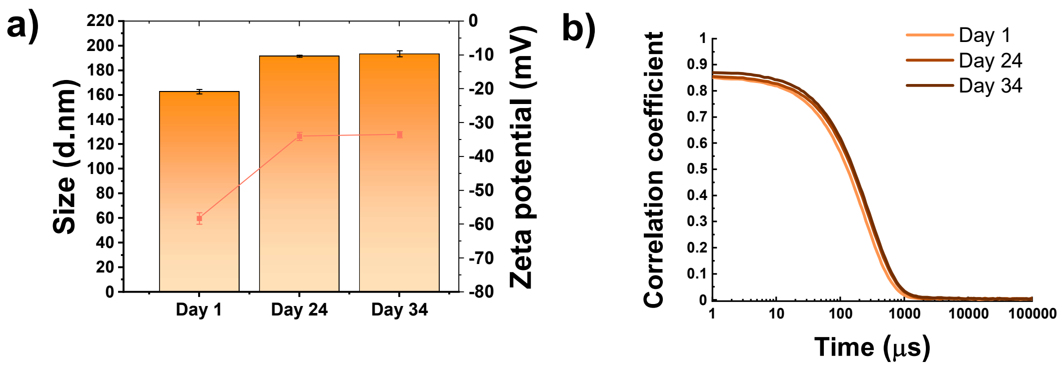

Figure S19. a) Stability up to 34 days indicating size and zeta potential for NPs obtained following approach 3. b) Corresponding correlograms after day 1, 24 and 34.

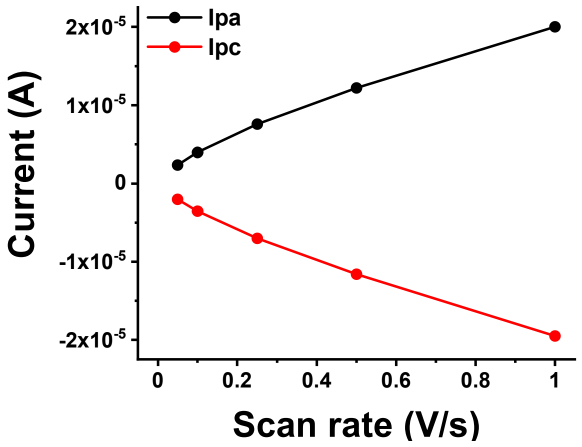

Figure S20. Current intensity versus scan rate from cyclic voltammetry experiments from one-pot surface modification to obtain EGaIn@SODPT-2.5DPPNT/Fc.
